# Opioid Use Disorder Documented at Delivery Hospitalization — United States, 1999–2014

**DOI:** 10.15585/mmwr.mm6731a1

**Published:** 2018-08-10

**Authors:** Sarah C. Haight, Jean Y. Ko, Van T. Tong, Michele K. Bohm, William M. Callaghan

**Affiliations:** ^1^Division of Reproductive Health, National Center for Chronic Disease Prevention and Health Promotion, CDC; ^2^Oak Ridge Institute for Science and Education, U.S. Department of Energy; ^3^United States Public Health Service, Commissioned Corps; ^4^Division of Unintentional Injury Prevention, National Center for Injury Prevention and Control, CDC.

## Abstract

Opioid use by pregnant women represents a significant public health concern given the association of opioid exposure and adverse maternal and neonatal outcomes, including preterm labor, stillbirth, neonatal abstinence syndrome, and maternal mortality (*1*,*2*). State-level actions are critical to curbing the opioid epidemic through programs and policies to reduce use of prescription opioids and illegal opioids including heroin and illicitly manufactured fentanyl, both of which contribute to the epidemic (*3*). Hospital discharge data from the 1999–2014 Healthcare Cost and Utilization Project (HCUP) were analyzed to describe U.S. national and state-specific trends in opioid use disorder documented at delivery hospitalization. Nationally, the prevalence of opioid use disorder more than quadrupled during 1999–2014 (from 1.5 per 1,000 delivery hospitalizations to 6.5; p<0.05). Increasing trends over time were observed in all 28 states with available data (p<0.05). In 2014, prevalence ranged from 0.7 in the District of Columbia (DC) to 48.6 in Vermont. Continued national, state, and provider efforts to prevent, monitor, and treat opioid use disorder among reproductive-aged and pregnant women are needed. Efforts might include improved access to data in Prescription Drug Monitoring Programs, increased substance abuse screening, use of medication-assisted therapy, and substance abuse treatment referrals.

Data were analyzed from the National Inpatient Sample (NIS; 1999–2014) and the State Inpatient Databases (SID; 1999–2014) of HCUP, Agency for Healthcare Research and Quality (*4*). NIS approximates a 20% stratified sample of all U.S. community hospital discharges participating in HCUP and is weighted to be nationally representative. Survey-specific analysis techniques were used to account for clustering, stratification, and weighting in NIS analyses (*4*). The SID contain state-specific data on hospital inpatient stays, regardless of payer; 30 states and DC had publically available data (Table). 

**TABLE:** National and state-specific prevalence of opioid use disorder per 1,000 delivery hospitalizations* — National Inpatient Sample (NIS)^†^ and State Inpatient Database,^§^ Healthcare Cost and Utilization Project (HCUP), 1999–2014

State	Year																Average annual rate change^¶^
	1999	2000	2001	2002	2003	2004	2005	2006	2007	2008	2009	2010	2011	2012	2013	2014	
**National**	**1.5**	**1.1**	**1.0**	**1.2**	**1.2**	**1.4**	**1.6**	**2.1**	**2.1**	**2.4**	**2.9**	**3.9**	**3.9**	**4.9**	**5.7**	**6.5**	**0.39**
Arizona	1.1	1.0	1.3	1.1	1.2	1.1	1.5	1.1	1.4	1.2	1.9	2.1	3.0	3.5	4.7	5.2	0.28
Arkansas	—	—	—	—	—	0.4	0.6	0.8	0.7	1.1	1.2	1.0	1.6	1.9	2.6	2.5	0.25
California	1.2	1.0	1.2	—	1.1	1.1	1.0	1.1	1.0	1.1	1.2	1.3	1.6	—	—	—	0.01
Colorado	0.4	0.5	0.6	0.6	0.7	0.7	0.7	0.8	1.0	1.1	1.2	1.6	1.8	2.1	2.9	3.6	0.20
District of Columbia	—	—	—	—	—	—	—	—	—	—	—	—	—	—	0.6	0.7	—**
Florida	0.5	0.5	0.5	0.6	0.7	0.9	1.0	1.2	1.6	2.1	3.0	4.1	5.1	5.6	6.3	6.6	0.58
Georgia	—	—	—	—	—	—	—	—	—	—	—	—	—	2.0	2.4	2.7	0.39
Hawaii	—	0.6	0.6	0.5	0.5	0.5	0.3	1.0	0.4	0.8	0.7	0.5	0.9	1.1	1.3	2.4	0.09
Iowa	0.1	0.2	0.0	0.0	0.1	0.2	0.2	0.2	0.2	0.4	0.5	0.6	0.8	1.2	1.4	1.3	0.10
Kentucky	—	0.4	0.9	1.6	2.4	2.5	3.1	3.9	4.0	5.1	6.1	7.2	9.5	14	15.7	19.3	1.55
Maine	0.7	0.6	1.5	2.3	4.0	—	—	9.4	10.7	13.5	21.7	26.2	27.8	34.1	—	—	4.13
Maryland	8.2	6.7	7.6	7.4	7.5	7.5	7.6	7.6	7.1	6.9	7.7	8.8	9.1	10.9	11.8	11.7	0.27
Massachusetts	2.0	2.7	2.4	2.6	2.9	3.7	4.6	4.9	6.5	6.9	8.3	8.8	9.6	12.2	13.1	—	0.90
Michigan	—	1.0	0.9	1.1	1.1	1.3	1.6	1.7	2.3	2.9	3.3	4.2	5.1	5.4	6.2	7.7	0.55
Minnesota	—	—	—	—	—	—	—	—	—	—	—	—	—	—	—	4.4	—**
Mississippi	—	—	—	—	—	—	—	—	—	—	—	1.9	1.6	—	—	—	—**
Nebraska	—	—	0.2	0.1	0.1	0.4	0.4	0.2	0.2	0.4	0.3	0.2	0.3	1.1	0.9	1.2	0.08
Nevada	—	—	—	0.6	0.6	1.2	1.1	1.1	1.0	1.3	1.7	2.0	2.3	3.1	3.4	4.5	0.33
New Jersey	4.1	4.3	4.0	3.8	4.0	3.4	4.0	3.3	3.5	3.6	4.1	4.5	4.5	5.0	5.3	5.6	0.08
New Mexico	—	—	—	—	—	—	—	—	—	3.8	3.9	5.5	7.6	10.6	13.6	14.8	2.47
New York	1.6	1.5	1.4	1.5	1.6	1.6	1.4	1.6	1.7	1.9	2.1	2.3	3.0	3.1	4.2	4.9	0.20
North Carolina	—	0.2	0.3	0.6	0.7	0.7	1.1	1.3	1.3	1.8	2.5	2.8	3.7	4.9	6.4	7.8	0.64
Oregon	1.2	0.9	1.5	1.7	1.4	1.8	2.1	2.5	2.1	2.7	3.8	4.4	4.4	5.7	6.9	8.4	0.49
Rhode Island	—	—	—	4.1	3.3	4.3	4.3	3.1	4.0	3.8	4.9	6.1	7.4	7.2	8.0	10.2	0.51
South Carolina	0.4	0.3	0.3	0.2	0.4	0.5	1.0	1.1	1.2	1.3	1.9	2.2	2.8	3.3	4.4	—	0.34
South Dakota	—	—	—	—	—	—	—	—	0.1	0.3	0.0	0.5	0.8	0.8	1.2	1.5	0.29
Utah	—	0.4	0.4	0.5	0.6	0.8	0.9	1.3	1.1	1.4	2.0	2.3	2.0	2.6	2.7	3.7	0.25
Vermont	—	—	0.5	2.4	3.7	4.0	7.6	12.9	14.6	19.0	28.5	27.1	33.8	43.7	51.1	48.6	5.37
Washington	1.2	0.9	1.1	1.3	1.7	1.9	2.4	2.8	2.6	3.4	4.2	5.3	6.9	7.1	8.5	10.8	0.71
West Virginia	—	0.6	1.0	1.6	2.3	3.0	4.2	6.8	7.1	8.2	10.1	11.2	15.3	21.3	29.8	32.1	2.83
Wisconsin	0.3	0.5	0.3	0.5	0.5	0.7	1.0	1.1	1.4	2.0	2.8	3.5	4.6	5.6	6.9	7.6	0.65

* Prevalence numerator consisted of cases of opioid type dependence and nondependent opioid abuse based on *International Classification of Diseases, Ninth Revision* (ICD-9) codes (304.00–304.03, 304.70–304.73, 305.50–305.53), and denominator consisted of national and state delivery hospitalization discharges.

^†^ Includes data from all states participating in HCUP each year (https://www.hcup-us.ahrq.gov/partners.jsp?NIS), weighted to produce national estimates. Rates through 2011 are weighted with trend weights, and rates 2012 and after are weighted using original NIS discharge weights to account for the change in NIS design in 2012.

^§^ Includes 30 states and the District of Columbia with publically available data. Availability of data ranged from 14 states in 1999 to 28 states in 2011.

^¶^ Estimated average annual change in the prevalence rate of opioid use disorder diagnoses per 1,000 delivery hospitalizations; all estimates were significant (p<0.05).

** Insufficient data (<3 years) to assess linear trend.

The annual number of in-hospital delivery discharges were identified from the 1999–2014 NIS and SID files using *International Classification of Diseases, Ninth Revision, Clinical Modification* (ICD-9-CM) diagnostic and procedure codes pertaining to obstetric delivery (*2*). Cases of opioid use disorder were identified from diagnoses of opioid dependence (ICD-9-CM 304.00–304.03, 304.70–304.73) and nondependent opioid abuse (ICD-9-CM 305.50–305.53), aligning with *Diagnostic and Statistical Manual-5* criteria.* Annual prevalence of opioid use disorder per 1,000 delivery hospitalizations during 1999–2014 was calculated nationally using NIS. Opioid use disorder prevalence was calculated using the SID for all 30 states and DC. For the 28 states with at least 3 consecutive years of data,^†^ linear trends were assessed using logistic regression. For states with significant trends (p-values <0.05), average annual rate changes were estimated from the beta coefficient for year and the national or state-specific intercept. A sensitivity analysis was performed to assess whether results differed in a resident-only sample.

During 1999–2014, the national prevalence of opioid use disorder increased 333%, from 1.5 cases per 1,000 delivery hospitalizations to 6.5 (Figure 1), an average annual increase of 0.4 per 1,000 delivery hospitalizations per year (p<0.05). State data were available for 30 states and DC; however, availability by year ranged from 14 states in 1999 to 28 states in 2011 (Table). In 1999, the prevalence of opioid use disorder ranged from 0.1 per 1,000 delivery hospitalizations in Iowa to 8.2 in Maryland, and in 2014, prevalence ranged from 0.7 in DC to 48.6 in Vermont; prevalence exceeded 30 per 1,000 delivery hospitalizations in Vermont and West Virginia (Figure 2). During 1999–2014, all 28 states experienced significant increasing linear trends (p<0.05) (Table). Over the study period, the average annual rate increase was lowest in California (0.01 per 1,000 delivery hospitalizations per year), whereas the highest average annual rate increases occurred in Maine, New Mexico, Vermont, and West Virginia, ranging from 2.5 to 5.4 opioid use disorder diagnoses per 1,000 delivery hospitalizations per year. The sensitivity analysis revealed no large differences between state residents and nonresidents.

**FIGURE 1:**
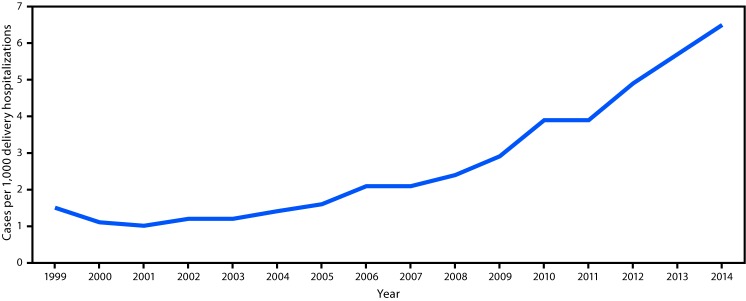
National prevalence of opioid use disorder per 1,000 delivery hospitalizations* — National Inpatient Sample (NIS),**^†^** Healthcare Cost and Utilization Project (HCUP), United States, 1999–2014 * Prevalence numerator consisted of cases of opioid type dependence and nondependent opioid abuse based on *International Classification of Diseases, Ninth Revision* (ICD-9) codes (304.00–304.03, 304.70–304.73, 305.50–305.53), and denominator consisted of delivery hospitalization discharges. ^†^ Includes data from all states participating in HCUP each year (https://www.hcup-us.ahrq.gov/partners.jsp?NIS), weighted to produce national estimates. Rates before 2012 are weighted with trend weights, and rates after 2012 are weighted using original NIS discharge weights to account for the change in NIS design in 2012.

**FIGURE 2:**
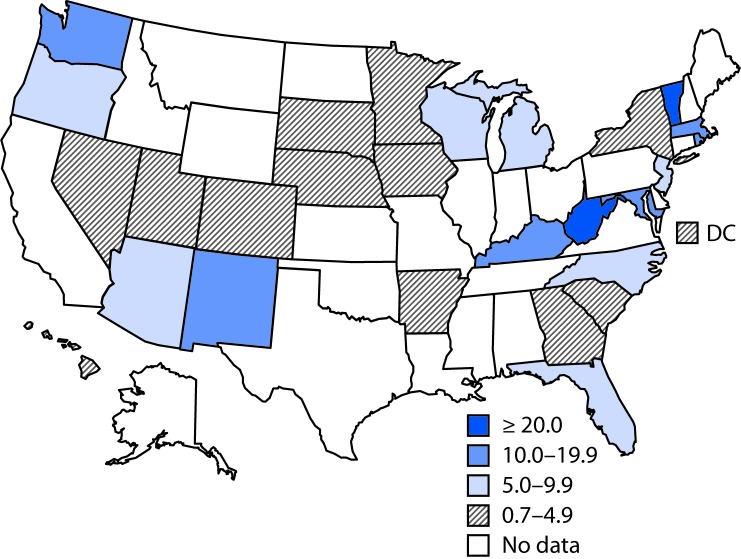
Prevalence of opioid use disorder per 1,000 delivery hospitalizations* — State Inpatient Database, Healthcare Cost and Utilization Project, 28 states, 2013–2014**^†^** * Prevalence numerator consisted of opioid type dependence and nondependent opioid abuse based on *International Classification of Diseases, Ninth Revision* (ICD-9) codes (304.00–304.03, 304.70–304.73, 305.50–305.53), and denominator consisted of state delivery hospitalization discharges. ^†^ Prevalence reported are for 2014, except for two states (Massachusetts and South Carolina) for which 2014 data were not available; 2013 data are reported for these states.

## Discussion

Nationally, rates of opioid use disorder at delivery hospitalization more than quadrupled during 1999–2014. These findings are consistent with previously documented national trends in opioid use disorder at delivery hospitalization during 1998–2011 (*2*) and increased national incidence of neonatal abstinence syndrome during 1999–2013 (*1*). Among 25 states and DC with 2014 data, the prevalence in Vermont and West Virginia was >3%. Although no previous multistate analyses of opioid use disorder at delivery hospitalization exist, these trends are mostly consistent with state neonatal abstinence syndrome estimates during 1999–2013 (*5*). Increasing trends might represent actual increases in prevalence or improved screening and diagnosis (*6*). Diagnostic procedures differ by state, and states with enhanced procedures for identifying infants with neonatal abstinence syndrome might ascertain more cases of maternal opioid use disorder.

These estimates also correlate with state opioid prescribing rates in the general population. West Virginia, for example, had a prescribing rate estimated at 138 opioid prescriptions per 100 persons in 2012, suggesting that individual persons might receive more than one opioid prescription per year (*7*). Excessive prescribing and challenges in accessing nonopioid treatments to control pain contribute to the rise in opioid use disorder. In an attempt to address prescribing rates, CDC supports maximizing and enhancing Prescription Drug Monitoring Programs, state-based databases that collect, monitor, and analyze controlled substance dispensing to detect risky prescribing practices and patient behaviors, such as multiple sources of prescriptions (*7*).

The 2016 CDC *Guideline for Prescribing Opioids for Chronic Pain* recommends that providers take an active role in combatting the opioid epidemic by considering opioid therapy for chronic pain only if expected benefits for pain and function are anticipated to outweigh risks (*8*). CDC and the American College of Obstetricians and Gynecologists (ACOG) guidelines recommend that before prescribing opioids for chronic pain, clinicians should ensure they are appropriate, review the Prescription Drug Monitoring Program, provide contraception counseling, and discuss risks of opioid use in pregnancy (*8*,*9*). ACOG recommends universal substance use screening at the first prenatal visit to manage opioid use disorder (*9*). If a patient has opioid use disorder, clinicians should prescribe medication-assisted therapy when possible and appropriate (*8*,*9*). Pregnant women with opioid use disorder involving heroin might require referral to harm reduction services (e.g., comprehensive syringe services). Arranging for pregnant patients with opioid use disorder to deliver at facilities prepared to monitor and care for infants with neonatal abstinence syndrome can facilitate access to appropriate care (*8*,*9*). After delivery, women might need referrals to postpartum psychosocial support services, substance-use treatment, and relapse-prevention programs (*8*).

Differing state policies might contribute to the state-to-state variability in opioid use disorder diagnosis. As of July 2018, eight states require health care professionals to test for prenatal drug exposure if it is suspected, and 24 states and DC require the reporting of suspected use (*10*). In addition, 23 states and DC consider substance use during pregnancy to be child abuse under child-welfare statutes, and three consider it grounds for civil commitment, which might result in women concealing substance use from their providers (*10*). However, data on the impact of these policies are scarce.

The findings in this report are subject to at least five limitations. First, not all states provide data to the public-use SID database. Within the data provided, not all hospitals participated; however, at least 80% of births reported to CDC’s National Center for Health Statistics are represented for each state.^§^ For the NIS, 2014 data were sampled from 45 states that include 94% of U.S. community hospital discharges. Second, analysis includes all hospital deliveries, regardless of the mother’s state of residency. Thus, results can only be interpreted for delivery hospitalizations in each state, which might not reflect trends among residents, although the sensitivity analysis revealed no large differences in rates by resident status. Third, results might not be generalizable to births that occurred outside of a hospital; these represent only 1.5% of all births.^¶^ Fourth, opioid use disorder might be underreported in this analysis; documentation of opioid use disorder at delivery hospitalization might not reflect diagnoses at other points in the pregnancy. Although universal verbal screening for substance use is recommended by ACOG (*9*), it is often not standard practice, which can lead to underestimates. Fifth, these data are ICD-code–dependent, limiting the ability to differentiate the source of opioid use disorder. The accuracy of codes might vary by hospital and state, leading to misreporting of opioid use disorder.

This first multistate analysis of opioid use disorder among delivery hospitalizations can be used by states to monitor the prevalence of opioid use disorder at delivery hospitalizations. There is continued need for national, state, and provider efforts to prevent, monitor, and treat opioid use disorder among reproductive-aged and pregnant women.

**Summary**

**What is already known about this topic?**

National rates of opioid use disorder are increasing among reproductive-aged and pregnant women, and opioid use during pregnancy is associated with adverse maternal and neonatal outcomes.

**What is added by this report?**

National opioid use disorder rates at delivery more than quadrupled during 1999–2014. Rates significantly increased in all 28 states with 3 years of data. Rate increases in Maine, New Mexico, Vermont, and West Virginia exceeded 2.5 per 1,000 deliveries per year. In 2014, rates ranged from 0.7 (District of Columbia) to 48.6 (Vermont).

**What are the implications for public health practice?**

National, state, and provider efforts are needed to prevent, monitor, and treat opioid use disorder among reproductive-aged and pregnant women.

## Footnotes

* https://pcssnow.org/wp-content/uploads/2014/02/5B-DSM-5-Opioid-Use-Disorder-Diagnostic-Criteria.pdf.

† Arizona, Arkansas, California, Colorado, Florida, Georgia, Hawaii, Iowa, Kentucky, Maine, Maryland, Massachusetts, Michigan, Nebraska, Nevada, New Jersey, New Mexico, New York, North Carolina, Oregon, Rhode Island, South Carolina, South Dakota, Utah, Vermont, Washington, West Virginia, and Wisconsin.

§ https://wonder.cdc.gov/natality.html.

¶ https://www.cdc.gov/nchs/data/nvsr/nvsr64/nvsr64_12.pdf.
